# Salvage use of venetoclax-based therapy for relapsed AML post allogeneic hematopoietic cell transplantation

**DOI:** 10.1038/s41408-021-00437-z

**Published:** 2021-03-04

**Authors:** Maansi Joshi, Joselle Cook, Kristen McCullough, Ahmad Nanaa, Naseema Gangat, James M. Foran, Hemant S. Murthy, Mohamed A. Kharfan-Dabaja, Lisa Sproat, Jeanne Palmer, Animesh Pardanani, Ayalew Tefferi, Kebede Begna, Michelle Elliot, Aref Al-Kali, Mrinal Patnaik, Mithun V. Shah, William J. Hogan, Mark R. Litzow, Hassan B. Alkhateeb

**Affiliations:** 1 Division of Hematology, Department of Internal Medicine, Mayo Clinic, Rochester, MN USA; 2 Division of Hematology & Medical Oncology, Department of Medicine, Mayo Clinic, Jacksonville, FL USA; 3 Division of Hematology, Department of Internal Medicine, Mayo Clinic, Scottsdale, AZ USA

**Keywords:** Health care, Molecularly targeted therapy

## Dear Editor,

Allogeneic hematopoietic cell transplantation (allo-HCT) is the only potentially curative treatment option available for patients with acute myeloid leukemia (AML) or myelodysplastic syndromes (MDS). Despite advances in optimizing conditioning regimens, graft-vs-host-disease (GVHD) management and supportive care, post-transplant relapse remains the Achilles heel impeding long-term survival^1^. Availability of effective therapies for these patients is an urgent area of unmet need.

BCL-2 is an anti-apoptotic protein and is present on leukocytes including leukemia stem cells. BCL-2 inhibitor venetoclax (VEN) has changed the treatment paradigm of AML in patients deemed unfit for intensive chemotherapy especially in frontline setting. Pollyea et al.^2^ showed the synergistic effect of azacitidine and venetoclax in selectively inhibiting oxidative phosphorylation in leukemia stem cells which disrupts the energy metabolism resulting in cellular death. A phase III trial by DiNardo et al.^3,4^ recruited elderly newly diagnosed AML patients (≥65 years) unfit for standard induction therapy. They showed a response rate of approximately 70% when treated with VEN in combination with a hypomethylating agent (HMA), a significant improvement on previously reported response rates of 20–30% with HMA alone^5–7^.

Although a prospective study is yet to be conducted, a moderate benefit has been suggested by retrospective studies in relapsed or refractory AML patients treated with VEN (or VEN-based regimens). A report of 43 patients treated with VEN in combination with HMA or low dose cytarabine (LDAC) demonstrated an overall response rate of 21% with 6-month overall survival of 24%^8^. In another study of VEN + HMA in 33 patients with r/r AML, 13 of whom had previously undergone an allo-HCT, the overall response rate was 64% with 50% CR + CRi and 1-year overall survival of 53%. The response rate in patients who underwent a prior allo-HCT was 46% (6 out of 13 patients)^9^. The utility of VEN in the post-transplant setting remains poorly studied. Here, we report on our cohort of patients who underwent VEN-based therapy for post-allograft relapse.

After institutional review board approval, we conducted a retrospective analysis on consecutive patients with relapsed AML post allo-HCT who received VEN-based therapy in the salvage setting within the 3-site Mayo Clinic Cancer Center (Minnesota, Arizona, and Florida). Comprehensive disease and transplant history were recorded. Patients were risk stratified and response was reported according to the ELN 2017 guidelines^10^. Overall survival was defined from the date of initiation of venetoclax to date of death from any cause. Leukemia-free survival was calculated from date of initiation of venetoclax to date of disease progression or death. Statistical analysis and Kaplan–Meier curves were computed using JMP 14 software.

Between December 2017 and April 2020, 29 patients (52% female) received VEN-based therapy for post-transplant relapse (Table 1). The median age at diagnosis was 58 years (range 20–72 years). Eleven patients (38%) had de novo AML, 5 patients (17%) had secondary AML post antecedent hematological disorder, 3 patients (10%) had therapy-related disease, and 10 patients (35%) had high-risk MDS. Twenty patients (69%) had adverse risk cytogenetics, eight (28%) had intermediate risk cytogenetics, and one had inversion 16. Molecular next-generation sequencing (NGS) data was available in 28 of 29 patients. *TP53* mutation or deletion was seen in 12 patients (41%) either at diagnosis or at relapse. In 7 patients, no pathogenic mutations were detected by NGS, they however had intermediate or adverse cytogenetic profile as per ELN classification. Other pathogenic mutations included epigenetic mutations (*ASXL1*, *TET2*, *IDH1* & *2*, *DNMT3A, SETBP1*), RNA splicing mutations (*SF3B1, SRSF2*), transcription factor mutations *(RUNX1, ETV6*), activated signaling mutations (NRAS, JAK2), and tumor suppressor mutations (*TP53, PHF6*) (details in Supplementary Table 1). At transplant, 20 patients were in CR1, 5 in CR2, 3 had persistent disease, and one patient had upfront allo-HCT for MDS. Fourteen patients received reduced intensity conditioning allo-HCT; the remaining 15 had myeloablative conditioning regimens. The donor source was matched unrelated donor (*n* = 17), matched related donor (*n* = 8), haploidentical (*n* = 2), and umbilical cord (*n* = 2). All patients received tacrolimus or cyclosporine plus methotrexate or mycophenolate-based GVHD prophylaxis, and four patients received ATG. Five patients had active GVHD at AML relapse, 2 with grade 1–2 GVHD were on steroids only, and 3 with grade 3–4 GVHD needed steroids with tacrolimus. The median follow-up was 16 months (range 2–50 months) post transplant.

**Table 1:** Baseline characteristics and response in patients salvaged with venetoclax.

Baseline characteristic	Total *n* = 29	Responders	Non responders	*P* value (Fisher’s exact test)
Age	58 years (20–72 years)	58 years (20–64 years)	57 years (25–72)	0.08
*Sex*				0.2
Male	14 (48%)	7 (50%)	7 (50%)	
Female	15 (52%)	4 (27%)	11 (73%)	
*AML type*				0.8
De novo	11 (38%)	5 (45%)	6 (55%)	
Secondary*	5 (17%)	2 (40%)	3 (60%)	
t-AML	3 (10%)	1 (33%)	2 (67%)	
High-risk MDS	10 (35%)	3 (30%)	7 (70%)	
*Cytogenetic risk group*				0.7
Favorable (inv 16)	1 (3%)	0	1 (100%)	
Intermediate	10 (35%)	4 (36%)	7 (64%)	
Adverse	18 (62%)	7 (39%)	11 (61%)	
*TP53 deletion or mutation*				0.7
Yes	12 (41%)	4 (33%)	8 (67%)	
No	17 (59%)	7 (41%)	10 (59%)	
Median no. of therapies prior to transplant	2 (range 1–4)	2 (range 1–4)	2 (range 0–3)	
*AML/MDS status at transplant*				0.1
CR1	20 (69%)	8 (47%)	9 (53%)	
CR2	5 (17%)	3 (60%)	2 (40%)	
Persistent disease or MRD positive	3 (11%)	0	3 (100%)	
Upfront transplant	1 (3%)	0	1 (100%)	
Time to relapse post transplant	9 months (range 2–37 months)	15 (range 2–37 months)	7.5 (range 2–35)	0.4
Time to venetoclax initiation from transplant	13 months (range 2–48)			
*AML status at venetoclax initiation*				0.8
Relapse 1 post transplant	21 (72%)	8 (38%)	13 (62%)	
Relapse 2 post transplant	4 (14%)	2 (50%)	2 (50%)	
Relapse 3 and/or refractory disease	4 (14%)	1 (25%)	3 (75%)	
Median no. of venetoclax cycles	2 (range 1–8)			
*Prior HMA exposure*				0.4
Yes	12 (41%)	3 (25%)	9 (75%)	
No	17 (59%)	8 (47%)	9 (53%)	

*AML secondary to underlying hematological disorder; *t-AML* therapy-related AML, *CR* complete remission, *MRD* minimal residual disease by flow cytometry.

Median time to relapse from transplant was 9 months (range 2–37 months). Twenty-one patients received VEN at first relapse post transplant, four were in second relapse, and four were in third relapse or had refractory disease. Five (17%) of 29 patients had extramedullary relapse in addition to bone marrow relapse and two of those patients had CNS disease. Prior exposure to HMA and VEN was seen in 11 patients and 1 patient, respectively. Median number of VEN cycles was 2 (range 1–10). The median duration of cycle 1 was 27 days (range 1–67 days; *n* = 29)), median cycle 2 duration was 27.5 days (15–63 days; *n* = 12), and of cycle 3 was 28 days (range 3–148 days; *n* = 4). All 29 patients required VEN dose adjustments due to concomitant antifungal prophylaxis with azoles due to inhibition of CYP3A-mediated metabolism^11^. In 26 patients VEN was given with either decitabine (*n* = 18) for 5 days or azacitidine (*n* = 8) for 7 days at standard doses. One patient received VEN with concomitant low dose cytarabine, one patient received gilteritinib, and one patient had VEN monotherapy. The 2 patients with CNS disease had concomitant IT chemotherapy. Of the 3 patients able to complete 8 or more cycles, VEN dosing was shortened to 21 days of 28-day cycle (Pt 21), 14 days of a 28-day cycle (Pt 22), and one patient (Pt 25) had their decitabine changed to 3 days instead of 5 days to mitigate the hematologic toxicity. Cycle details are fully outlined in the Supplementary Tables 1 and 2. The proposed VEN dosing schedule was 28 days for all patients with MDS (*n* = 10); treatment details were evaluable for 9 patients. The median cycle 1 length was 32 days (range 8–67 days); four patients went on to cycle 2, median cycle length was 47.5 days (range 21–59), and two patients completed cycle 3 whose median length was 45 days (range 3–148).

**Fig. 1:**
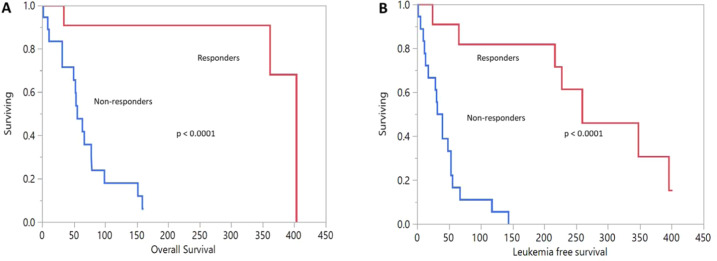
Salvage use of venetoclax-based therapy for relapsed AML post allogeneic hematopoietic stem cell transplant. **A** Overall survival and **B** leukemia-free survival in responders vs non-responders in patients receiving VEN for post-transplant relapse.

The overall response rate was 38% (*n* = 11) with eight patients (28%) achieving complete remission (CR/CRi) and one a partial remission (PR) and two patients had a reduction in blast count. Median time to CR was 2.5 months (range 1–4 months) after a median of 2 cycles (range 1–3 cycles) to achieve best response. Median duration of response in those who responded was 7 months (range 1–11 months). Out of five patients with extramedullary disease, two (40%) achieved CR, one with CNS disease and one with testicular and skin disease. Median overall survival after VEN initiation was 79 days (range 2–403 days) (Supplementary Table 3). Overall survival in responders vs non-responders was 403 days (95% CI 361–403) vs 55 days (95% CI 32–78) (log rank *p* < 0.0001). Median leukemia free survival (LFS) in responders was 259 days (95% CI 65–395) and in non-responders was 35 days (95% CI 13–53) (log rank *p* < 0.0001) (Fig. 1). Four of 12 patients (33%) with *TP53* mutation responded, 3 achieved CR and one patient had PR. One *TP53* mutated patient in CR went on to have a second allograft and is currently in remission. There was, however, no difference in overall survival between those with *TP53* mutation vs wild type (*p* = 0.8). At last follow up, 4 patients continue to be in CR, 2 still on VEN, one post second alloHCT, and one post high-dose cytarabine (HIDAC) and IT chemotherapy. Twenty-three patients progressed on VEN and received additional therapy or transitioned to hospice, and in 2 patients the response was unknown or not evaluable.

Grade 3 or 4 toxicities were neutropenia (*n* = 20), infections (*n* = 16), thrombocytopenia (*n* = 19) with diffuse alveolar hemorrhage seen in 2 patients, and anemia (*n* = 15). One patient had a confirmed invasive fungal infection (pulmonary aspergillosis) despite azole prophylaxis, ten patients had culture-positive bacterial infections, and 5 patients had culture-negative febrile neutropenia. Nineteen patients required cycle length adjustments due to hematological toxicity. This included shortening duration of VEN therapy to 14–21 days and/or delaying subsequent cycles. There were no instances of GVHD flare while on VEN.

Treatment options for post-transplant relapse are often dictated by how ‘robust’ the patient is. Although clinical trials are always considered the preferred approach, often these patients are excluded, rendering their options limited to donor lymphocyte infusion (DLI), a second allo-HCT, or high-dose chemotherapy (HDCT)^12^. Notably, DLI has limited applicability in the setting of active GVHD, and patient’s performance score is generally a limitation to pursuing a second allo-HCT owing to the resulting toxicities resulting from the allo-HCT. Chemotherapy, although associated with higher toxicity, results in higher response rates compared with HMA alone^13^. In our cohort VEN-based therapy yielded an ORR of 38%, and prolonged both OS and LFS in responders. We noticed modest durable responses to this therapy with some activity in both *TP53*-mutated patients and those with extramedullary disease. Other groups have shown similar results with VEN use in high-risk patients^14,15^. A common challenge with VEN-based therapy is identifying the appropriate dose and cycle duration due to hematological toxicity. In our cohort, we noted most clinicians reduced duration of VEN and/or delayed subsequent treatment cycles when necessary. We were unable to determine the optimum dose to achieve best response while minimizing toxicity. Despite the small sample size and retrospective nature of our study, we provide evidence that even in the context of post-transplant relapse with adverse mutations or extramedullary disease, VEN-based therapy is capable of inducing CR and improving survival in responding patients. Future prospective studies focusing on appropriate patient selection and to define dose and duration of therapy are needed in this patient population.

## Supplementary information

supplementary
