# Supplementary material for: Salvage use of venetoclax-based therapy for relapsed AML post allogeneic hematopoietic cell transplantation
Source: Blood Cancer J. 2021 Mar 4;11(3):49. doi: 10.1038/s41408-021-00437-z (PMC7933161; doi:10.1038/s41408-021-00437-z)
Supplement: Supplementary file 1 — supplementary [file 41408_2021_437_MOESM1_ESM.docx]

**Supplementary**

**Salvage use of Venetoclax-based therapy for relapsed AML post allogeneic hematopoietic cell transplantation**

Maansi Joshi*^1^, Joselle Cook*^1^, Kristen McCoullough^1^, Ahmad Nanaa^1^, Naseema Gangat^1^, James M. Foran^3^, Hemant S Murthy^3^, Mohamed A. Kharfan-Dabaja^3^, Lisa Sproat^2^, Jeanne Palmer^2^, Animesh Pardanani^1^, Ayalew Tefferi^1^, Kebede Begna^1^, Michelle Elliot^1^, Aref Al-Kali^1^, Mrinal Patnaik^1^, Mithun V Shah^1^, William J Hogan^1^, Mark R Litzow^1^, Hassan B Alkhateeb^1^

^1^*Division of Hematology, Department of Internal Medicine, Mayo Clinic, Rochester, MN, USA*

^2^*Division of Hematology, Department of Internal Medicine, Mayo Clinic, Scottsdale, AZ, USA*

^3^*Division of Hematology & Medical Oncology, Department of Medicine, Mayo Clinic, Jacksonville, FL, USA*

**Supplementary Table 1: Baseline characteristics and treatment data for the entire cohort.**

| **PT** | **Age** | **Diagnosis** | **Karyotype at diagnosis** | **ELN cytogenetic risk** | **Molecular abnormality** | **No. of therapy lines prior to transplant** | **Type of transplant** | **Type of relapse** | **Disease status at VEN initiation** | **No of cycles of VEN** | **Dose VEN** | **HMA/ Companion drug** | **Antifungal** | **Cycle comments** | **BR on VEN** | **LFS**  **Days** | **OS days** |
| --- | --- | --- | --- | --- | --- | --- | --- | --- | --- | --- | --- | --- | --- | --- | --- | --- | --- |
| 1 | 64 | MDS EB | 43-45,XYYc,add(4)(q31),-5,add(7)(q21),-8,-22,+1~3mar[cp5]/47,XYYc[20] | Adverse | SF3B1 12%, TP53 26% | 3 | RIC / MUD | BM | Relapse 1 | 5 | 100 | decitabine | POSA |  | CR | 216 | 403 |
| 2 | 30 | De novo AML | 46,XY,t(10;17)(p13;q21)[18]/46,XY[2] | Intermediate | None | 1 | MAC / Haplo | BM | Relapse 1 | 1 | 400 | Decitabine (10 day) | VORI |  | NE | 5 | 53 |
| 3 | 31 | De novo AML | 47,XY,t(10;11)(p12;q13),+21[1]/49,XY,t(10;11) | Intermediate | NRAS | 2 | MAC / double cord | BM | Relapse 1 | 1 | 100 | decitabine | POSA |  | PD | 17 | 32 |
| 4 | 50 | Secondary AML | 46,XX,inv(3)(q21q26.2),der(6)t(6;6) (p11.2;q23)[10]/45,idem,-7(3)/46,XX[7]. | Adverse | BCOR 18%, SF3B1 20% | 3 | MAC / MUD | BM | Relapse 1 | 2 | 100 | AZA | POSA |  | PD | 53 | 54 |
| 5 | 20 | De novo AML | 46,XY,t(6;11)(q27;q23),add(12)(p13)[2]/46,XY[4] | Intermediate | None | 4 | MAC / MUD | Testes + Skin | Relapse 1 | 1 | 100 | decitabine | VORI then POSA | Venetoclax decreased from 400mg to 200mg after cycle 2 with addition of Posaconazole | CR | 110 | 110 |
| 6 | 58 | De novo AML | 48,XX,+13,del(17)(p11.2),+19[10] | Adverse | RUNX1 35%, TP53 32% | 4 | MAC / MUD | BM | Relapse 1 | 4 | 300 | Decitabine and Cytarabine | FLUC | Cycle dates NA | PR | 65 | 84 |
| 7 | 67 | Secondary AML | 43,X,-Y,add(3)(q23),del(5)(q13q33),-7,-12,-15,add(16)(q24),del(17)(p11.2),+mar[12]/46,XY[8]1 | Adverse | TP53 | 0 | RIC / MUD | BM | Relapse 1 | 4 | 100 | Decitabine | POSA |  | PD | 143 | 152 |
| 8 | 25 | Secondary AML | 50,XX,+X,+5,+6,+20[17]/46,XX[3 | Adverse | None | 3 | MAC / MUD | CNS + BM | Relapse 1 | 1 | 200 | Decitabine | FLU |  | NE | 29 | 29 |
| 9 | 55 | MDS EB | 46,XX,add(3)(q27)[7]/46,XX[13] | Intermediate | None | 2 | MAC /MUD | BM | Relapse 1 | 4 | 400 | AZA | VORI |  | PD | 117 | 159 |
| 10 | 65 | De novo AML | 46,XY | Adverse | ASXL1 35%, IDH1 38%, SRSF2 43%, TET2 40%, DNMT3A 42%, RUNX1 37% | 2 | RIC / MRD | BM | Relapse 1 | 1 | 200 | Decitabine | FLUC | Dose reduced from 200mg to 100mg cycle 1 day 22 | MLFS | 24 | 34 |
| 11 | 69 | MDS EB | 46,XX,del(5)(q13q33),del(11)(q13q23)[9]/46,XX[11] | Adverse | ASXL1 31%, CBL 70%, SETBP1 17% | 1 | RIC / MUD | BM | Relapse 2 | 1 | 200 | Decitabine | POSA |  | PD | 31 | 50 |
| 12 | 21 | De novo AML | 46,XX,t(6;11)(q27;q23)[11]/51,sl,+3,+der(6)t(6;11),+8,+18,+19[8]/46,XX[1] | Adverse | NRAS | 3 | MAC / MRD | CNS+BM | Relapse 1 | 4 | 100 | AZA | POSA |  | CR (with concomitant IT chemo) | 401 | 401 |
| 13 | 64 | MDS EB | 46,XY,add(1)(p36.1),add(2)(p23),del(5)(q13q33),add(17)(q23)[14]/46,XY[6] | Adverse | BCOR 10%, RUNX1 7% | 3 | RIC / MUD | BM | Relapse 1 | 2 | 100 | Decitabine | POSA |  | PD | 53 | 161 |
| 14 | 70 | De novo AML | 40-46,XX,-3,add(4)(p16),-5,add(6)(q12),add(7)(p11.2),der(9)t(9;10)(p22;q11.2),-10,der(14;15)(q10;q10),-17,-18,-20,-22,add(22)(q13),+2-6mar[cp20] | Adverse | TP53 59%, KRAS 34% | 2 | RIC / MUD | BM | Relapse 1 | 1 | 100 | Decitabine | POSA |  | NE | 1 | 2 |
| 15 | 68 | Therapy related AML | 46,XY,t(17;17)(q11.2;q25)[2]/ 46,sl,add(7)(p13)[2]/46,XY[16] | Adverse | TP53 | 3 | RIC / MRD | BM | Relapse 1 | 2 | 200 | Decitabine | FLUC | Stopped for profound cytopenia | PD | 48 | 79 |
| 16 | 46 | MDS EB | 44,XY,t(2;13)(q35;q14),add(3)(q11.1),-5,-7,-13,-17,i(17)(q10),+2mar[2]/46,XY,del(7)(q22q34)[1]/45,X,-Y[6]/46,XY[11] | Adverse | TP53 | 2 | MAC / MUD | BM + EM (pelvic sarcoma) | Relapse 2 | 1 | 100 | AZA | POSA |  | PD | 9 | 9 |
| 17 | 37 | Therapy related AML | 44-47,XX,t(2;21)(q31;q22),der(4;18)(q10;p10),-5,add(5)(q11.2),+idic(8)(p11.2),+add(8)(p11.2),add(8)(p21),der(8;13)(q10;q10),add(9)(p22),der(12)t(1;12)(p32;p13),-17,-18,-22,+0-1r,+0 -2mar[cp13]/84-92,XXXX,t(2;21)(q31;q22)x2,der(4;18)(q10;p10)x2,add(5)(q11.2)x2,+idic(8)(p11.2)x2,add(8)(p11.2),der(8;13) (q10;q10)x2,der(12)t(1;12)(p32;p13)x2,-17,-17,-22,-22,+1-5mar[cp7] | Adverse | TP53 88% | 1 | MAC / MRD | BM | Relapse 1 | 1 | 200 | Decitabine | FLUC |  | PD | 39 | 64 |
| 18 | 58 | MDS EB | 46,XY,del(5)(q15q33),-6,idic(6;6)(q11;q11),del(7)(q22q34),del(12)(p11.2p13),add(16)(q12),-18,-20,+21,-22,+2-5mar[cp20] | Adverse | TP53 16% | 3 | RIC / MUD | BM | Relapse 1 | 1 | 200 | Decitabine | FLUC |  | PD | 13 | 99 |
| 19 | 72 | De novo AML | 47,XY,+8[7]/46,XY[13] | Intermediate | DNMT3A 48%, FLT3 5%, IDH1 45%, NRAS 22%, PHF6 94% | 1 | MAC / MRD | BM | Relapse 1 | 2 | 200 | Gilteritinib | FLUC |  | PD | 55 | 56 |
| 20 | 53 | De novo AML | 47,XX,del(7q)(q32),+13,inv(16)(p13.1q22)[19]/46,XX[1] | Favorable | None | 2 | MAC / MUD | BM | Relapse 3 | 1 | 100 | AZA | POSA |  | NE | 11 | 11 |
| 21 | 57 | Therapy related AML | 46,XY,del(20)(q11.2q13.3)[17]/46,sl,t(3;17)(p21;q25)[3] | Intermediate | DNMT3A 9%, BCOR 19%, SF3B1 10%, IDH1 37% | 1 | MAC / MRD | BM | Relapse 1 | 10 | 100 | AZA | POSA | Starting cycle 3, azacitadine given 5 days, and venetoclax for 21 days of a 28 day cycle for cytopenias. From Cycle 8, VEN duration decreased to 14 days of a 28 day cycle, AZZA dose reduced by 50%  ** GVHD | CR | 242 | 242 |
| 22 | 60 | Secondary AML | 47,XX,+19[11]/48,idem,+8[3]/46,XX[6] | Intermediate | None | 1 | RIC / MRD | BM | Refractory | 8 | 100 | AZA | VORI | From Cycle 3 VEN switched to 14 days of a 28 day cycle for ccytopenia | PR | 259 | 307 |
| 23 | 54 | Secondary AML | 46,XX,del(7)(q21q36)[16]/46,XX[4] | Adverse | CSF3R 25%, DNMT3A 27%, IDH2 27%, PHF6 31% | 1 | RIC / MUD | BM | Relapse 2 | 7 | 100 | AZA | VORI | Venetoclax given continuously 28 day cycle | CR | 227 | 246 |
| 24 | 51 | MDS EB | 46,XX,i(17)(q10)[20] | Adverse | ASXL1 30%, SRSF2 49%, TP53 | 2 | MAC / Haplo | BM | Refractory | 2 | 200 | NONE | POSA |  | PD | 67 | 78 |
| 25 | 29 | De novo AML | Not available | Adverse | TP53 | 4 | RIC / MUD | BM | Relapse 1 | 10 | 100 | DEC | POSA | From Cycle 7, Decitabine duration reduced to 3 days for cytopenias | CR | 228 | 228 |
| 26 | 63 | MDS EB | 43-48,X,-Y,del(4)(p14p15),del5(q13),add5(q31),-7,der(7;14)(q10;q10),-8,der(9;14)(q10;q10),add(14)(p11.2),-17,-18,add19(p13.1),-20,+21,+22,+r1,+r2,+mar1,+mar2,+mar3,+mar[cp16]/94-96,idemx2,-7,+7,add(14)x2,+3-4mar[cp2]/46,XY[3] | Adverse | TP53 6% | 1 | RIC / MUD | BM | Relapse 1 | 5 | 400 | Cytarabine | FLUC |  | CR | 395 | 395 |
| 27 | 62 | MDS EB | Not available | Adverse | None | 1 | RIC / MRD | EM | Relapse 1 | 1 | 300 | Decitabine | POSA |  | PD | 39 | 67 |
| 28 | 64 | MDS EB | 46,XY | Intermediate | Not performed | 1 | MAC / Cord | BM | Relapse 2 | 6 | 200 | Decitabine |  | Missing cycle dates | CR | 347 | 361 |
| 29 | 60 | De novo AML | 44,XX,inv(2)(p13q21),+add(5)(q11.2),add(5)(q11.2),i(8)(q10),-17,-18,, -19, add (21))(p11.2)[16]/45,XX,add(4)(q12),+add(5)(q11.2),add(5)(q11.2),+8, add(8)(p11.2),i(8)(q10),-17,-18,-19,add(21)(p11.2)[3]/46,XX[1] | Adverse | TP53 51% | 1 | RIC / MUD | BM | Refractory | 1 | 100 | Decitabine | POSA |  | PD | 28 | 32 |

*Where available, VAF has been included for molecular abnormalities.

ELN: European Leukemia Net; OS: Overall survival; LFS: leukemia free survival; VEN: Venetoclax; MDS EB: myelodysplastic syndrome with excess blasts; AML: acute myeloid leukemia; RIC: reduced intensity conditioning; MAC: myeloablative conditioning; MUD: matched unrelated donor; MRD: matched related donor; Haplo: haploidentical transplant; BM: bone marrow; CNS: central nervous system; CR: complete remission; PR: partial remission or blast reduction; MLFS: morphologic leukemia free survival; PD: persistent or progressive disease; NE: not evaluable.; VORI: Voriconazole; POSA: Posaconazole; FLUC: Fluconazole;

**Supplementary Table 2: VEN cycle details**

| Cycle no | No of evaluable patients | Median cycle length (range) |
| --- | --- | --- |
| 1 | 29 | 27(1-67) |
| 2 | 12 | 27.5 (15-63) |
| 3 | 7 | 28 (3-148) |
| 4 | 4 | 28 (24-28) |
| 5 | 3 | 28(29-35) |
| 6 | 3 | 28(28-42) |
| 7 | 2 | 32.5 (28-37) |
| 8 | 2 | 61.5 (42-81) |
| 9 | 2 | 31 (28-34) |
| 10 | 2 | 31 (27-35) |

**Supplementary Table 3: Survival in patients treated with VEN for post-transplant relapse**

| **Survival** | **Days** | **P value** |
| --- | --- | --- |
| **Median Overall Survival**   - Responders - Non-responders | 79 days (range 2 – 403 days)  403 days (95% CI 361 - 403)  55 days (95% CI 32 – 78) | P < 0.001 |
| **Median Leukemia free survival**   - Responders - Non-responders | 53 days (range 1 – 403)  259 days (95% CI 65 – 395)  35 days (95% CI 13 – 53) | P < 0.001 |
